# Development of a Multiplex TaqMan Assay for Rapid Detection of Groundnut Bud Necrosis Virus: A Quarantine Pathogen in the USA

**DOI:** 10.3390/v17040532

**Published:** 2025-04-05

**Authors:** Anushi Suwaneththiya Deraniyagala, Avijit Roy, Shyam Tallury, Hari Kishan Sudini, Albert K. Culbreath, Sudeep Bag

**Affiliations:** 1 Department of Plant Pathology, University of Georgia, Tifton, GA 31793, USA; 2 Molecular Plant Pathology Laboratory, Beltsville Agricultural Research Center (BARC), Unites States Department of Agriculture (USDA)-Agricultural Research Service (ARS), Beltsville, MD 20705, USA; 3 Plant Genetic Resources Conservation Unit (PGRCU), United States Department of Agriculture (USDA)-Agricultural Research Service (ARS), Griffin, GA 30223, USA; 4 International Crops Research Institute for the Semi-Arid Tropics, Patancheru, Hyderabad 502324, Telangana, India

**Keywords:** groundnut bud necrosis orthotospovirus (GBNV), *Orthotospovirus arachinecrosis*, phytosanitary measures, TaqMan RT-qPCR, quarantine pathogen

## Abstract

Groundnut bud necrosis orthotospovirus (GBNV), a tripartite single-stranded RNA virus, poses a significant threat to United States agriculture. GBNV is a quarantine pathogen, and its introduction could lead to severe damage to economically important crops, such as groundnuts, tomatoes, potatoes, peas, and soybeans. For the rapid and accurate detection of GBNV at points of entry, TaqMan reverse transcriptase–quantitative polymerase chain reaction (RT-qPCR) assays were developed and the results validated using conventional reverse transcriptase–polymerase chain reaction (RT-PCR) followed by Sanger sequencing. These assays target highly conserved regions of the nucleocapsid (NP) and movement (MP) proteins within the viral genome. Multiplex GBNV detection assays targeting the NP and MP genes, as well as an internal control plant gene, ACT11, showed efficiency rates between 90% and 100% and R^2^ values of 0.98 to 0.99, indicating high accuracy and precision. Moreover, there was no significant difference in sensitivity between multiplex and singleplex assays, ensuring reliable detection across various plant tissues. This rapid, sensitive, and specific diagnostic assay will provide a valuable tool at ports of entry to prevent the entry of GBNV into the United States.

## 1. Introduction

Groundnut bud necrosis virus (GBNV; *Orthotospovirus arachinecrosis*), also known as peanut bud necrosis virus, was first described in India [1]. GBNV is a negative-strand RNA virus, one of the members of the genus *Orthotospovirus* and family *Tospoviridae* [2,3]. Its enveloped, quasi-spherical virion measures approximately 80–110 nm in diameter and possesses a tripartite genome consisting of small (S), medium (M), and large (L) segments of single-stranded RNA [4]. The L RNA is a negative-sense RNA that encodes the viral polymerase [5]. The M RNA segment is ambisense, encodes the non-structural movement protein (NSm) in sense direction, and is a precursor to the two viral membrane glycoproteins Gn and Gc in a complementary sense. The S RNA encodes the virus sense non-structural protein (NSs) and the complementary sense nucleocapsid (NP) protein [6,7].

The major hosts of GBNV include cowpeas, mung beans, soybeans, potatoes, and tomatoes, but it can infect oil seeds, vegetables, ornamentals, and weeds [8,9], with global crop losses of 89 million USD [10]. The virus induces a variety of symptoms in peanuts, such as chlorosis, necrosis, seedling wilt, stunting, altered size, and discoloration of pods and seeds [11,12]. Tospoviruses are transmitted by thrips species in the order Thysanoptera, and the major vectors of GBNV are *Frankliniella schultzei* (common blossom thrips), *Scirtothrips dorsalis* (chili thrips), and *Thrips palmi* (melon thrips) [13].

GBNV is presently restricted to a few Asian countries, including Bangladesh, China, India, Indonesia, Iran, Nepal, Sri Lanka, and Thailand [12,14]. However, the major host plants and all three known GBNV thrips vectors are present in the United States. This raises significant concern, as some of these vectors are highly efficient at transmitting other orthotospoviruses, like tomato spotted wilt orthotospovirus (*Orthotospovirus tomatomaculae*, TSWV) and groundnut ring spot orthotospovirus (*Orthotospovirus arachianuli*, GRSV), to valuable crops in the US [12,14,15].

Extensive research has been conducted on GBNV, encompassing molecular characterization and diversity analysis [16,17,18,19], infection mechanisms [20], the interactions of GBNV with the host/vector [21,22], and symptom development [9,20]. Several assays have been employed for the detection of GBNV, like ELISA [16,19], reverse transcriptase–polymerase chain reaction (RT-PCR) [6,9,17,18,21,22], and SYBR Green-based qPCR [20]. Very recently, a reverse transcriptase-loop-mediated isothermal amplification (RT-LAMP) assay was developed for the specific GBNV detection of potato stem necrosis disease; it was found to be more sensitive than a conventional RT-PCR assay [23]. Despite the adequacy of the current methods for routine diagnosis, a more sensitive and specific gold-standard assay is indispensable to safeguard against the involuntary entry of GBNV into countries currently unaffected by the virus. In this study, we developed and validated a TaqMan-based, highly specific, and sensitive multiplex reverse transcriptase–quantitative polymerase chain reaction (RT-qPCR) diagnostic assay for GBNV detection, targeting two different open reading frames (ORFs) encodes NP and MP genes along with the plant internal control actin gene (ACT11).

## 2. Materials and Methods

### 2.1. Collection of Infected Plant Materials

Two infected peanut (*Arachis hypogaea* L.) leaf samples were procured from India. The first one was collected from research fields at the International Crops Research Institute for the Semi-Arid Tropics (ICRISAT) in Hyderabad. The second was an isolate maintained on cowpeas (*Vigna unguiculata*) at the Indian Agricultural Research Institute (IARI) in New Delhi. Both samples were freeze-dried and mailed to the plant virology lab, UGA Tifton, under USDA-APHIS-PPQ permit #PCIP-20-00223.

To perform a specificity test, peanut leaf samples infected with TSWV were collected from the UGA research farm in Tifton and maintained on *Nicotiana tabacum*. Nucleocapsid protein (NP) and movement protein (MP) genes for tomato chlorotic spot orthotospovirus (TCSV; *Orthotospovirus tomatoflavi*) and GRSV were artificially synthesized (GenScript, NJ, USA) based on the conserved sequences available in NCBI GenBank (available on the September 2019). Peanut plants of SunOleic 97R [24] grown in an insect-free growth chamber were used as healthy controls.

### 2.2. Total Plant RNA Extraction and RNA Assessment

The total RNA was extracted from 40 mg of freeze-dried tissue and ground in liquid N_2_ using the RNeasy Plant Mini Kit (Qiagen, Germantown, MD, USA), following the manufacturer’s instructions. The RNA was eluted in 40 µL of nuclease-free water; the quality and quantity were evaluated using a NanoDrop spectrophotometer (Thermo Fisher Scientific, Waltham, MA, USA). The total RNA was immediately diluted in RNase-free water to a final concentration of 50 ng/µL, aliquoted, and stored at −80 °C for further processing. To prepare DNase-treated RNA controls, the total RNA was treated with the TURBO DNA-free Kit (Invitrogen, San Diego, CA, USA), following the manufacturer’s instructions, before quantification using NanoDrop (Thermo Fisher Scientific).

### 2.3. Primer Design for Conventional RT-PCR and Primers and Probes for TaqMan RT-qPCR Assays

The primers for the conventional RT-PCR and TaqMan assays were designed to target conserved regions of nucleocapsid and movement protein genes encoded by the S and M RNAs of GBNV, respectively (Table 1). The primers and probes were designed based on the sequences available at NCBI-GenBank using MEGA-X [25] and BioEdit [26] software. The primers targeting the internal controls-β-actin (ACT11) of the peanut plant were adopted from [27]. The TaqMan probe (ACT1) for the peanut ACT11 gene was designed based on the sequences in NCBI-GenBank, which aligned within the specific primer pair. The designs of the primers and TaqMan probes were optimized in silico using the PrimerQuest (Integrated DNA Technologies, Coralville, IA, USA) and NCBI Primer-BLAST tools. The primers and probes for the TaqMan assay (with attached fluorophore–quencher pairs) were synthesized at Bio-Rad, Hercules, CA, USA). The primers used and the fluorescently labeled probes were made in 275 and 250 nmol-scale synthesis, respectively, and mixed in a 1:1 primer and probe ratio.

### 2.4. Conventional RT-PCR and Sanger Sequencing

The conventional RT-PCR assay for GBNV was carried out in two-step reactions. The first-strand cDNA was synthesized from 150 ng of total RNA using Superscript III reverse transcriptase (Invitrogen), following the manufacturer’s instructions. The total RNA (150 ng), 1 µL of dNTP mix (10 mM), 1 µL of gene-specific reverse primer (10 µM), and nuclease-free water (up to 11 µL) were mixed in PCR tubes and incubated at 65 °C for 5 min followed by one minute on ice for the initial annealing of the reverse primer. Four microliters of 5X First-Strand Buffer, 1 µL of DTT (100 mM), 1 µL of RNaseOUT Recombinant Ribonuclease Inhibitor (40 U/µL), and 1 µL of M-MLV Superscript III reverse transcriptase (200 U/μL) were added to the same tube and incubated under the following cycling conditions: inhibition of RNase A activity at 25 °C for 5 min, synthesis of cDNA at 55 °C for 1 h, and a final step of inactivation of reverse transcriptase at 70 °C for 15 min. To optimize the reaction protocols, the primers were initially tested with gradient PCR for the annealing step with a temperature range of 45 to 55 °C. The PCR master mix had a total volume of 25 µL, consisting of 2.5 µL of PCR buffer (10×), 0.75 µL of MgCl_2_ (50 mM), 0.5 µL of dNTP mix (10 mM), 0.5 µL of forward primer (10 µM), 0.5 µL of reverse primer (10 µM), 0.5 µL of Platinum Taq Polymerase (5 u/µL), 17.75 µL of nuclease-free water, and 2.0 µL of template cDNA.

The cycling parameter consisted of initial denaturation at 95 °C for 5 min followed by 35 cycles of 94 °C for 0.30 s, 52 °C (SB226F/R) or 43 °C (SB227F/R) for 1.00 min, and 72 °C for 1.00 min and a final extension step of 72 °C for 10 min. The PCR products were analyzed in 0.8% agarose gel electrophoresis, and the DNA was stained using GelRed Nucleic Acid Gel Stain (Biotium, Fremont, CA, USA). The amplified fragments of the expected sizes were purified using a QIAquick gel extraction kit (Qiagen), cloned in pGEM-T Easy Vector (Promega, Madison, WI, USA), and sequenced. Then, the sequences were analyzed using MEGA-X [25], BioEdit [26] software, and the NCBI-BLASTn tool (Genewiz, South Plainfield, NJ, USA).

### 2.5. TaqMan Reverse Transcription-Quantitative PCR (RT-qPCR)

The 4X Reliance One-Step Multiplex Supermix (Bio-Rad) was used for all single and multiplex TaqMan RT-qPCR assays in one-step reactions. The reactions were set in 20 µL and contained 1 µL of each primer–probe mix (PrimePCR. Bio-Rad) and 100 ng of RNA. The cycling conditions were 10 min at 50 °C for the cDNA synthesis and 10 min at 95 °C for the polymerase activation/initial denaturation, followed by 35 cycles for 0.10 s at 95 °C and 0.30 s at 53 °C. A gradient test was carried out for the annealing step from 53 to 63 °C to select the optimum annealing temperature (Tm) in Biorad T100 Thermal cycler. The optimal annealing temperature for the multiplexing was selected based on the singleplex and multiplex assay gradient fluorescence data. The analyses of the fluorescence data were conducted using the thermocycler CFX Maestro software 2.3 (Biorad, Hercules, CA, USA).

Multiplex RT-qPCRs including three primer–probe pairs (GBNV NP, GBNV MP, and ACT11) were run under the same cycling conditions for singleplex reactions, except the reaction mix included the primers and probes for all three of the regions targeted. For the non-reverse transcriptase controls, 4X Reliance One-Step Multiplex Supermix (Bio-Rad) was incubated at 95 °C for 1–2 min to inactivate reverse transcriptase. For the controls, healthy DNase-treated peanut plant RNA (50 ng/µL) was added. Efficiency comparisons were performed for each targeted region, comparing the Cq values obtained for the multiplex and singleplex assays for six replicates. The Wilcoxon signed-rank test was conducted using JMP software (JMP^®^, Version 14. SAS Institute Inc., Cary, NC, USA, 1989–2019).

### 2.6. Standard Curves

To evaluate the efficiency of the reactions, standard curves were constructed using the total RNA extracted from a peanut leaf sample infected with GBNV after verification by TaqMan RT-qPCR. Fifteen samples were prepared, each with a twofold dilution, using a starting amount of 200 ng of total RNA. The efficiency (E) and R2 values of each standard curve were calculated by the Bio-Rad CFX Maestro software 2.3 using the following equation: (E = (10 (–1/slope)1) × (100)).

### 2.7. External Validation

To confirm the reproducibility of the developed protocols, the Standard Operating Procedure (SOP) for the assay was shared with ICRISAT-India for validation study using the positive GBNV controls and the suspected GBNV-like symptomatic samples from the fields.

## 3. Results

The outcomes of this research are summarized below in different sections, with the selection of primers and probes, development of the conventional RT-PCR, and singleplex and multiplex RT-qPCR assays for GBNV detection and their correlations.

### 3.1. In Silico Analysis for Primer and Probe Selection

For the primer design, 54 NP and 47 MP complete GBNV sequences, available in NCBI GenBank, were aligned and analyzed to identify the conserved regions using BioEdit and MEGA X software. Specific annealing temperatures and other important parameters, like the self-complementarity and 3’-self complementarity values of the designed amplicon, were analyzed using Primer-BLAST in NCBI.

### 3.2. Development of Conventional RT-PCR Assay for Detection of GBNV

**Figure 1:**
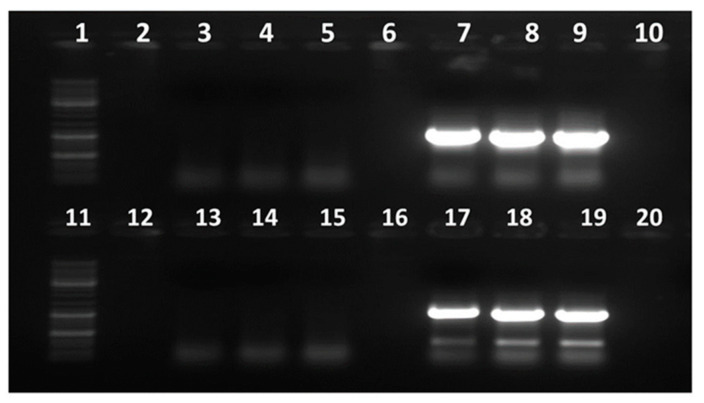
Gel electrophoresis showing RT-PCR amplifications of GBNV NP and MP.

The presence of GBNV was confirmed in the total RNA (50 ng/µL) extracted from the Delhi and ICRISAT isolates by amplifying the 1046 bp S RNA and 1119 bp M RNA amplicons encoding the NP (SB226F/R) and MP (SB227F/R) genes, respectively (Figure 1). The amplified products were cloned, sequenced, and submitted to NCBI GenBank and received the following accession numbers: OL469156, OL469157, OL469158, and OL469159. Each total RNA (50 ng/µL), extracted from either peanut leaf samples infected with TSWV or from *N. tabacum* infected with TSWV, was used as a negative control.

Wells 1–10 show the GBNV MP (~1.1 kb) and controls: Well 1—DNA Ladder (Gene Ruler 1 kb Plus DNA Ladder, Thermo Scientific); Wells 2 and 10—empty wells (no sample loaded); Well 3—non-template control (NTC) (ensures no contamination in PCR); Well 4—healthy peanut plant RNA (a negative control for GBNV MP); Well 5—TSWV-infected peanut plant RNA (another negative control to confirm no cross-species amplification, as TSWV and GBNV both belong to the Orthotospovirus genus); and Wells 7–9—GBNV MP (~1.1 kb) (expected bands indicate successful amplification of the GBNV MP).

Wells 11–20 show the GBNV NP (~1.05 kb) and controls: Well 11—DNA Ladder (GeneRuler 1 kb Plus DNA Ladder, Thermo Scientific); Wells 12 and 20—empty wells (no sample loaded); Well 13—non-template control (NTC) (ensures no contamination in PCR); Well 14—healthy peanut plant RNA (a negative control for GBNV NP); Well 15—TSWV-infected peanut plant RNA (another negative control to confirm no cross-species amplification, as TSWV and GBNV both belong to the Orthotospovirus genus); and Wells 17–19—GBNV NP (~1.05 kb) (expected bands confirm successful amplification of GBNV NP).

### 3.3. Development of Singleplex TaqMan RT-qPCR Assay

Two primer–probe sets specifically for GBNV MP, GBNV NP, and ACT11 amplified the target through a temperature range of 53–63 °C. The temperature range was selected based on each calculated and working annealing temperature (Tm). The lowest Cq values obtained at 53 °C for the GBNV MP; the GBNV NP; and the internal control gene, ACT11, in the GBNV-infected peanut plants were 23.72 ± 0.557, 21.58 ± 0.429, and 26.17 ± 0.041, respectively, and 53 °C was selected as the annealing temperatures. The ACT11 (internal control gene) Cq value for the healthy peanut plants was observed to be 23.66 ± 0.171. To confirm the specificity, RNA extracted from peanut leaf samples or *N. tabacum* infected with TSWV and synthesized NP and MP genes for TCSV and GRSV were used as negative controls No amplification was observed in negative controls.

### 3.4. Efficiencies, Sensitivities, and Detection Limits of the Singleplex Reactions

**Figure 2:**
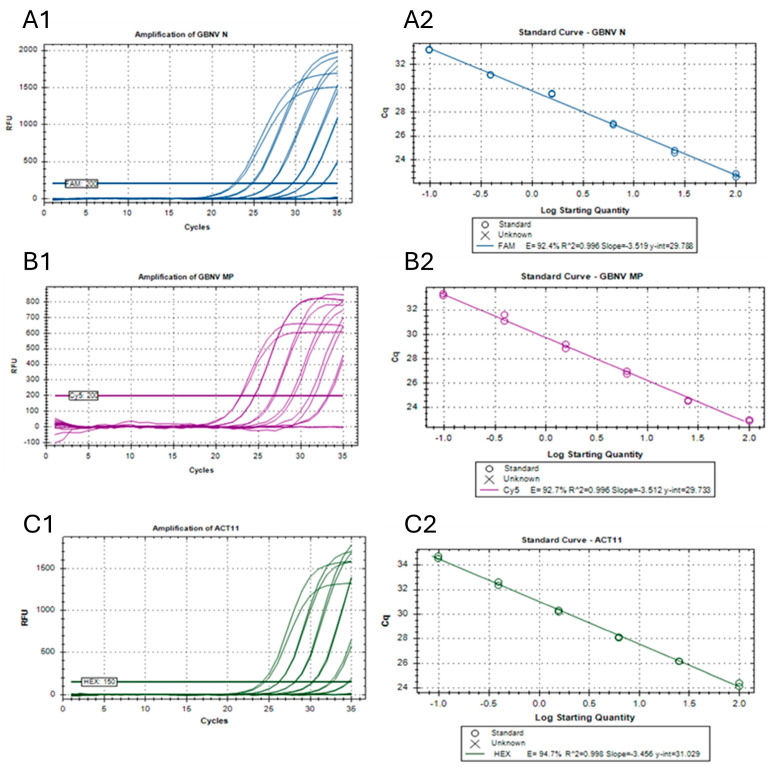
(**A1**–**C2**) Observation of efficiency and sensitivity of the singleplex reactions in TaqMan real-time quantitative reverse-transcription PCR.

Each reaction efficiency (E) was observed, and a coefficient of determinations (R2) was calculated at 53 °C for each singleplex reaction (Figure 2). Standard curves were obtained by twofold dilution series of the total RNA, which consisted of 15 dilutions (Table 2). For the primers targeting the GBNV NP, the E value was 91.6% and R2 was 0.997, while for the GBNV MP, E and R2 were 90.6 and 0.995, respectively. The E value was observed as 94.1% and R2 was 0.998 for the standard curve for the internal control, ACT11, at 53 °C. The Cq determination mode was always set to a single-threshold mode. The experiment reaction efficiency (E) was within the recommended range of 90–110%, with R2 ≥ 0.99, suggesting that these assays are reliable for the detection of GBNV.

The primer–probe combinations for the GBNV NP (GBNV_NF/R and GBNV_N) and GBNV MP (GBNV_MPF/R and GBNV_MP) were able to detect the virus from a total RNA amount of as little as 0.098 ng (means: Cq = 34.48 ± 0.045 for GBNV NP and 34.45 ± 0.009 for GBNV MP). The minimal detectable amount of total RNA for the primer–probe combination for the internal control, ACT11 (ACT1F/R and ACT1), was as low as 0.195 ng (Figure 2C1 and Table 2).

The fluorophores (with designated colors) for the targeted GBNV NP, GBNV MP, and actin genes were FAM (dark blue), Cy5 (purple), and Hex (dark green), respectively. When the total plant RNA amount (plant RNA + virus RNA) was within the range of 200 ng–0.098 ng in the 20 µL reaction mix, the Cq values were observed to be in between 22.73 ± 0.204 and 34.48 ± 0.045 for the GBNV NP (Figure 2A1) and 22.95 ± 0.081 and 34.45 ± 0.009 for the GBNV MP (Figure 2B1), respectively. The lowest Cq value for the actin gene in 200 ng of the total RNA was 24.28 ± 0.206, while the highest Cq value (34.63 ± 0.141) was recorded for 0.195 ng of the total RNA (Figure 2C1). Using the amplification data of the twofold dilution series of the total RNA (Figure 2A1,B1,C1), standard curves were obtained for the GBNV NP (Figure 2A2), GBNV MP (Figure 2B2), and actin (Figure 2C2) genes using Bio-Rad CFX Maestro software.

### 3.5. Development of TaqMan-Based Multiplex RT-qPCR

**Figure 3:**
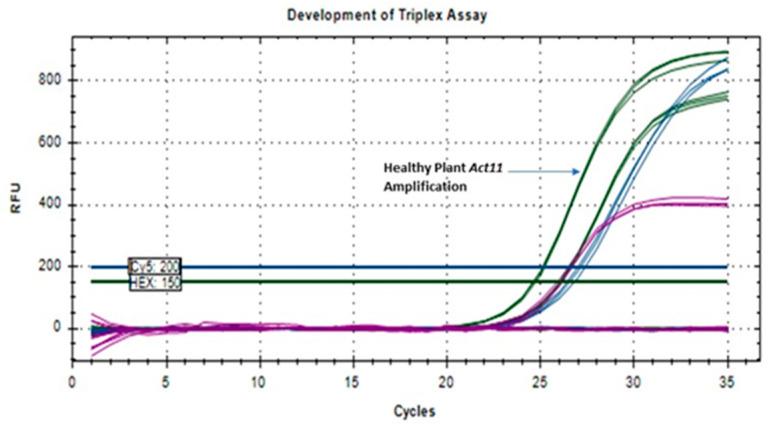
Standardization of multiplex real-time RT-PCR assays. The fluorophores (with designated colors) for the targeted GBNV NP, GBNV MP, and actin genes were FAM (dark blue), Cy5 (purple), and Hex (dark green), respectively.

Successful amplification of the GBNV NP and MP and plant ACT11 genes was observed (Figure 3) at an annealing temperature of 53 °C in a one-step multiplex reaction. The Cq values for the GBNV NP and GBNV MP were 27.18 ± 0.189 and 26.61 ± 0.115, respectively. The ACT11 showed Cq values of 26.05 ± 0.060 in infected samples and 24.64 ± 0.078 in healthy samples. Amplification was observed for the GBNV NP with the fluorophore FAM (indicated in dark blue), the GBNV MP with the fluorophore Cy5 (indicated in purple), and ACT11 with the fluorophore Hex (indicated in dark green; Figure 3). To confirm the specificity, RNA extracted from peanut leaf samples or *N*. *tabacum* infected with TSWV, as well as synthesized NP and MP genes for TCSV and GRSV, were used as negative controls. No amplification was observed for any of these samples.

### 3.6. Comparisons of Efficiency of Singleplex and Multiplex RT-qPCR Assays

The quantification cycle (Cq) values in both the multiplex and singleplex assays (Table 3) were compared using the Wilcoxon signed-rank test (α = 0.05). The *p* values obtained for the GBNV NP, the GBNV MP, and ACT11 were 0.0625, 0.7188, and 0.0313 (Table 4). The differences in the Cq values were insignificant for both the GBNV NP and MP regions (∴ *p* > 0.05). The difference was highly insignificant for the GBNV MP compared to the GBNV NP. The Cq values obtained for the multiplex and singleplex assays for ACT11 (Table 3) were found to be significantly different (∴ *p* < 0.05) (Table 4).

### 3.7. Specificity of the TaqMan Assay Developed for GBNV

The primer–probe sets designed for GBNV, targeting the NP (GBNV-NF/R and GBNV_N) and MP (GBNV-MPF/R and GBNV_MP) genes, did not amplify the corresponding genomic regions of other closely related orthotospoviruses (TSWV, TCSV, and GRSV). The samples containing GBNV showed high fluorescence signals (GBNV NP: Cq = 23.25 ± 0.149; GBNV MP: Cq = 21.35 ± 0.265), and there were no amplifications with the other orthotospoviruses tested. The viral genes did not amplify in the healthy controls. The primer–probe set designed for targeting the ACT11 (GBNV-NF/R and GBNV_N) gene amplified the corresponding genomic regions of the plant in the healthy plant RNA and in the virus-infected plant RNA. No amplifications were observed in the non-template controls (NTCs).

In the non-reverse transcriptase (NRT) controls, no amplification of the viral genes was observed. However, ACT11 gene amplification was observed in the GBNV-infected samples and healthy plant samples. The Cq value for ACT11 was lower in the NRT controls for the GBNV-infected peanut plants and higher for the healthy peanut plants. In the DNase-treated RNA controls, significantly higher Cq values were observed for the ACT11 gene compared with the non-treated controls in both the GBNV-infected and healthy peanut samples (Table 5). For the GBNV NP, the Cq values for the DNase-treated and non-treated controls were 23.40 ± 0.083 and 23.25 ± 0.149, respectively. For the GBNV MP, the Cq values for the DNase-treated and non-treated controls were 21.31 ± 0.401 and 21.35 ± 0.265, respectively.

### 3.8. External Validation of the Assay

The assay developed here was also validated at ICRISAT-India using the field samples. The samples included were GBNV-infected peanut and cowpea and healthy peanut leaf samples obtained from the greenhouse, along with a non-registered field sample. The assay was replicated flawlessly, and the pathogen was detected using the protocol provided (Table 6).

## 4. Discussion

This study aimed to develop a sensitive, specific, and innovative multiplex TaqMan qRT-PCR assay for the rapid detection of groundnut bud necrosis virus (GBNV) in plant materials. Recognized as a critical quarantine pathogen, the potential introduction of GBNV into the US could severely impact food production and ecological systems. Therefore, the rapid and reliable detection of GBNV is crucial for effective virological surveillance and timely decision-making [28]. By advancing surveillance and management strategies, this research seeks to safeguard agricultural health and biosecurity. As discussed, the assays used to diagnose GBNV are not very effective for the early detection of virus activity in samples [29] with low virus titers [29] or in samples where diagnostics are difficult, such as infected seeds or asymptomatic infected propagative materials [30].

In addition to RT-PCR [17] and serological assays like ELISA [19], the SYBR Green technique [20,21] has been successfully developed for the detection of GBNV NSs. In this study, we have developed three diagnostic assays based on conventional RT-PCR and RT-qPCR assays for the specific, rapid, and sensitive detection of GBNV.

The RT-PCR designed gives amplicons resulting in complete NP and MP, separately, which are present on S RNA and M RNA, respectively. SYBR Green can bind to any ds DNA, making it less specific and leading to overquantification of products. The use of TaqMan probes is better than SYBR Green due to being specific and highly sensitive [28]. In these assays, multiple regions were targeted to mitigate PCR bias, as the choice of primers and specific isolates can lead to significant differences in PCR amplification results [31,32,33,34,35,36,37,38,39,40]. The first multiplex RT-qPCR assay developed in this study targeted two different segments of GBNV’s segmented tripartite genome, significantly enhancing the assay’s capability to detect even recombinant strains of GBNV. This advancement addresses the genetic variations in the virus that can occur through mutation, recombination, and segment reassortment over time.

Quantitative PCR (qPCR) allows fast detection and quantification of the pathogen. However, the specificity depends upon the selected sequences of primers and the probe design [31,32,33,34]. With the rapid availability of inexpensive sequencing technologies, a vast amount of sequence data are available for viruses and other plant pathogens. The availability of these sequence data allows for the understanding of the design of the virus and its strains, specific primers, and probes. Also, these primers and probes can be designed to differentiate different strains or isolates [41].

In this study, primers and probes were designed, targeting conserved regions of GBNV MP and NP based on the sequences deposited in GenBank for accurate identification. For real-time quantification, an internal control plant gene, ACT11, was used, as the expression of ACT11 is relatively stable under abiotic and biotic stresses [27]. To reduce the labor, time, and cost, multiplex PCR/qPCR can be used to detect more than one targeted region; genes can be employed in detecting more than one species/strain/isolate in the same reaction tube. Multiplex qPCRs are currently being used to detect a number of viruses due to their fast, sensitive, quantitative, reproducible, and specific nature. Confirmed by the preliminary in silico analysis, with the compatible thermodynamics of the primer–probe sets, the TaqMan RT-qPCR technique was successfully implemented. The results show that the singleplex and multiplex assays allow the specific and sensitive detection of both the viral genes for GBNV MP and NP as well as the internal control plant gene. These new assays are very specific in detecting GBNV and nonspecific to the closely related orthotospovirus species TSWV, GRSV, and TCSV, minimizing the chance occurrence of false positives. Furthermore, this assay was highly sensitive and was able to detect GBNV in a total RNA of 0.098 ng.

When plants or plant products arrive at a port of entry, they are held in quarantine until it is confirmed that there is no risk of quarantine pest introduction. Quick detection will result in the rapid and safe release of the plants or plant materials [42]. In a conventional multiplex PCR, different targets are amplified and detected using gel electrophoresis, but in a multiplex RT-qPCR, differentiation is achieved using different fluorescence dyes and unaffected by amplicon size. Fluorescent dyes with different wavelengths are used to avoid cross-absorption, and thus, the efficiency is higher. In the multiplex assay developed in this study, there were no cross-interactions among the targets, and the statistical analyses comparisons confirmed that there were nonsignificant differences in the Cq values for the viral genes.

The multiplex protocol developed in our plant virology lab, UGA, Tifton was validated by ICRISAT-India. Protocol validation, or external validation, is an important process that involves conducting experiments or tests using the same protocol in multiple independent laboratories to ensure that the results obtained are consistent and reproducible across different settings. The data are supported by the reliability and generalizability of the protocol beyond its initial development. This also confirms the robustness of the protocol and increases the confidence in its applicability to a wider range of conditions.

Genetic variation in GBNV, which has a segmented tripartite genome, can occur through mutation, recombination, and segment reassortment [43]. In this study, we developed the first multiplex real-time RT-qPCR assay that targets two different segments, significantly enhancing its ability to detect even recombinant strains of GBNV. In conclusion, a sensitive, specific, and novel multiplex TaqMan qRT PCR assay for the rapid detection of GBNV in plant materials for post-entry quarantine purposes represents a valuable tool for global germplasm introduction or acquisition and the exchange of improved cultivars. Thus, it can be used for specific and rapid sensitive detection to determine active infection of GBNV in plant materials. This assay could be incorporated into Standard Operating Procedures (SOPs) and applied at key facilities, such as the US National Plant Germplasm Inspection Station, USDA-APHIS, and ICRISAT, including at ports of entry. It can be used to pre-screen commodities to detect GBNV-infected materials before export, thereby minimizing the risk associated with the spread of GBNV. This study is the first report of using RT-qPCR based assays for the detection of GBNV using multiple gene targets.

## Figures and Tables

**Table 1:** Primers and probes used in this study for assay development and validation.

RT-PCR Assay	Primer Name	Target	5′-3′ Sequence	Tm (C)	Amplicon Size (bp)	Reference
Conventional	SB226F	GBNV nucleocapsid protein (NP)	CAAGGACTTTCTGTGTTCC	52	1046	This study
	SB226R		AAGATTGCCTCTTTCGAGGTC			
	SB227F	GBNV movement protein (MP)	GAAATAATGTCTCGCTTTTCTAA	43	1119	This study
	SB227R		TTTCAAGAAGATTATCCATCTC			
TaqMan	GBNV-NF	GBNV NP	TTCCTAATTTCTCTTTCTTCACA	53	137	This study
	GBNV-NR		ATCTTTCGATACATGTGCTTTAA			
	GBNV_N		**6-FAM** AGGACCTCCAATGCAGAGCATCAT **Iowa Black FQ**			
	GBNV-MPF	GBNV MP	GAACTGGTGGGAAACAGATA	53	130	This study
	GBNV-MPR		ATTTCAAGAAGATTATCCATCTC			
	GBNV_MP		**Cy5** TCTCATCATCATTTTCAGCTTCTAAT **Iowa Black RQ-Sp**			
	ACT1F	βactin (ACT11)	ATGCTAGTGGTCGTACAACTGG	50–60	108	[27]
	ACT1R		CTAGACGAAGGATAGCATGTGG			
	ACT1		**Hex** TGGTGTCAGCCACACAGTCCCCAT **Iowa Black FQ**			This study

**Table 2:** Two fold RNA dilution series for minimal detection limit for primer-probe combination.

Standard	Concentration of Total RNA (ng/µL) in GBNV-Infected Dried Leaf Tissue	Total ng in 20 µL Reaction Mix	Cq Value for MP	Cq Value for NP	Cq Value for *ACT11*
1	100	200	22.95 ± 0.081	22.73 ± 0.204	24.48 ± 0.206
2	50	100	23.57 ± 0.006	23.51 ± 0.108	25.11 ± 0.061
3	25	50	24.56 ± 0.042	24.70 ± 0.152	26.17 ± 0.013
4	12.50	25	25.69 ± 0.733	25.89 ± 0.020	27.12 ± 0.057
5	6.25	12.5	26.86 ± 0.169	26.99 ± 0.086	28.10 ± 0.048
6	3.125	6.25	27.86 ± 0.008	28.03 ± 0.107	29.15 ± 0.013
7	1.563	3.125	29.01 ± 0.245	29.54 ± 0.050	30.25 ± 0.098
8	0.781	1.563	30.42 ± 0.258	29.88 ± 0.058	31.37 ± 0.073
9	0.391	0.781	31.34 ± 0.363	31.11 ± 0.012	32.48 ± 0.190
10	0.195	0.391	32.21 ± 0.105	32.15 ± 0.062	33.52 ± 0.111
11	0.098	0.195	33.25 ± 0.116	33.21 ± 0.036	34.63 ± 0.141
12	0.049	0.098	34.46 ± 0.009	34.48 ± 0.045	-
13	0.024	0.049	-	-	-
14	0.012	0.024	-	-	-
15	0.006	0.012	-	-	-

**Table 3:** The Cq values were obtained for each targeted region for the multiplex (triplex) and singleplex assays separately. Six replicates were considered for each comparison.

Targeted Region	Replicate	Cq Values	
		Multiplex	Singleplex
GBNV NP	1	21.18	20.98
	2	21.25	20.9
	3	21.22	20.87
	4	21.19	20.86
	5	21.21	21
	6	21.08	21.15
GBNV MP	1	22.04	21.97
	2	21.63	21.79
	3	21.93	21.7
	4	21.82	21.89
	5	21.38	21.55
	6	21.79	21.86
ACT11	1	27.08	27.04
	2	26.84	26.58
	3	26.91	26.77
	4	27.02	26.87
	5	27.12	26.76
	6	27.07	27.04

**Table 4:** Comparison of the Cq values between singleplex and multiplex assays for each region. The nonparametric Wilcoxon signed-rank test was conducted using JMP software (α = 0.05). *p* ≤ 0.05 is statistically significant and is represented with *.

Targeted Region	Cq Value (Singleplex)–Cq Value (Multiplex)	
	Test Statistic S	Prob > |S|
GBNV NP	−9.500	0.0625
GBNV MP	2.500	0.7188
ACT11	−10.500	0.0313 *

**Table 5:** Mean Cq values gained for additional two-comparison multiplex RT-qPCR assays.

Sample	Targeted Region	Non-Reverse Transcriptase (RT Was Inactivated)	Non-Treated (No DNAse Treatment or RT Inactivation Was Performed)	DNase-Treated
Healthy peanut RNA	ACT11	24.91 ± 0.229	23.90 ± 0.222	27.75 ± 0.074
GBNV-infected RNA	ACT11	24.88 ± 0.083	25.54 ± 0.238	30.72 ± 0.081
	GBNV NP	NA	23.25 ± 0.149	23.40 ± 0.083
	GBNV MP	NA	21.35 ± 0.265	21.31 ± 0.401

**Table 6:** Sample description and mean Cq values which represent the cycle number at which fluorescence signal crosses a threshold for multiplex RT-qPCR.

Sample	Targeted Region	Mean Cq Value
Healthy peanut RNA	ACT11	30.02 ± 0.164
	GBNV NP	NA
	GBNV MP	NA
GBNV-infected (cowpea)	ACT11	29.06 ± 0.172
	GBNV NP	19.99 ± 0.122
	GBNV MP	18.03 ± 0.276
GBNV-infected (peanut)	ACT11	28.56 ± 0.503
	GBNV NP	16.46 ± 0.444
	GBNV MP	15.26 ± 0.716
GBNV-infected (unknown from the field)	ACT11	25.86 ± 0.177
	GBNV NP	28.31 ± 0.130
	GBNV MP	25.56 ± 0.199

## Data Availability

Data are contained within the article.
